# What does time mean in development?

**DOI:** 10.1242/dev.164368

**Published:** 2018-06-26

**Authors:** Miki Ebisuya, James Briscoe

**Affiliations:** 1 RIKEN Center for Biosystems Dynamics Research (RIKEN BDR), 2-2-3 Minatojima-minamimachi, Chuo-ku, Kobe, 650-0047, Japan; 2 The Francis Crick Institute, 1 Midland Road, London NW1 1AT, UK

## Abstract

Biology is dynamic. Timescales range from frenetic sub-second ion fluxes and enzymatic reactions to the glacial millions of years of evolutionary change. Falling somewhere in the middle of this range are the processes we usually study in development: cell division and differentiation, gene expression, cell-cell signalling, and morphogenesis. But what sets the tempo and manages the order of developmental events? Are the order and tempo different between species? How is the sequence of multiple events coordinated? Here, we discuss the importance of time for developing embryos, highlighting the necessity for global as well as cell-autonomous control. New reagents and tools in imaging and genomic engineering, combined with *in vitro* culture, are beginning to offer fresh perspectives and molecular insight into the origin and mechanisms of developmental time.

## Abstract (precis)

**Summary:** What sets the tempo and manages the order of developmental events? New reagents and techniques in imaging, genomic engineering and *in vitro* culture are beginning to offer insight into the mechanisms of developmental time.

## Introduction

The importance of time is a tacit assumption in many accounts of developmental mechanisms. Timely cell division and differentiation is necessary for the growth and assembly of functional, well-proportioned tissues. Small variations produce the differences that distinguish individuals, whereas pronounced changes result in more major alterations in the timing or organisation of developmental processes that characterize differences between species. Moreover, abnormalities in timing can lead to defects in the assembly of tissues, resulting in dysfunction that can be incompatible with survival. Consequently, understanding how temporal information is encoded and read in developing systems is crucial for understanding the mechanisms of embryogenesis and evolutionary change. In specific cases, we know something about the mechanisms, but there is still a lot to discover and general principles remain unclear (Duboule, 2003; Johnson and Day, 2000; Reiss, 2003).

## The order and tempo of developmental processes

Events in a developing embryo occur in a particular sequence and ensuring the correct order is essential for a successful outcome. In addition, the speed of progression through a developmental sequence is important: it determines the overall duration of development and controls the rate of individual developmental processes. As embryos don't have access to an external clock or a timetable to regulate the order and tempo of development, the schedule must be generated by mechanisms within the embryo itself.

**Fig. 1.**
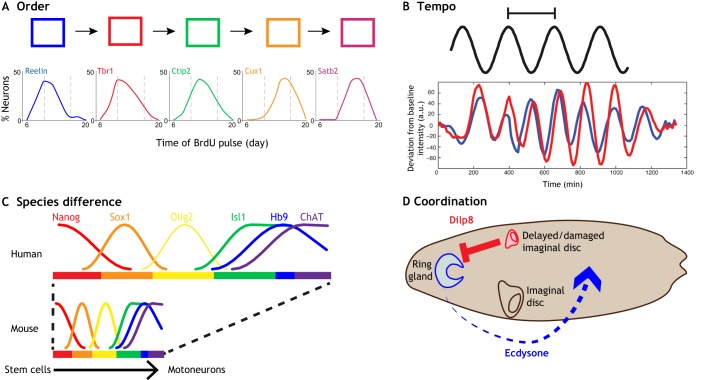
**Time plays an important role in development.** (A) The order of marker gene expression in embryonic stem-cell derived cortical neurogenesis. Reelin and Tbr1 are subplate or Cajal-Retzius neuron markers; Tbr1 and Ctip2 are deep layer neuron markers; Cux1 and Satb2 are upper layer makers. Redrawn from Gaspard et al. (2008). (B) Regular oscillations in gene expression in the pre-somitic mesoderm correlate with the rhythmic generation of somites. Red and blue lines represent the levels of a Lfng reporter in two neighbouring regions of tissue in cultured pre-somitic mesoderm, indicating in-phase synchronization. Adapted from Tsiairis and Aulehla (2016), where it was published under a CC-BY license (https://creativecommons.org/licenses/by/4.0/). (C) Motoneuron differentiation involves a series of changes in gene expression. These are identical in mouse and human but take different amounts of time. Nanog is a pluripotent stem cell marker; Sox1 is a neural progenitor marker; Olig2 is a motoneuron progenitor marker; Isl1, Hb9 and ChAT are terminal motoneuron markers. Reproduced from Davis-Dusenbery et al. (2014). (D) Developmental checkpoints coordinate the progression of *Drosophila* larvae. Ecdysone acts systemically to trigger pupariation and metamorphosis. The production of ecdysone can be delayed for several days if imaginal discs are damaged or their growth abnormal. An insulin-like peptide, Dilp8, is secreted from immature imaginal discs to block ecdysone production.

In many tissues, different cell types are produced in a stereotypical sequence from progenitor cells, such that the fate of the progeny depends on when they were born (Fig. 1A). Well-studied examples of this include the generation of different neuronal and glial subtypes in the *Drosophila* ventral nerve cord, the vertebrate retina and cerebral cortex (reviewed by Toma et al., 2016). In each case, the changes in cell type generation are driven by changes in the gene expression programme of progenitors. In the *Drosophila* nerve cord, four transcription factors – referred to as temporal identity factors – are activated and repressed in turn, and are both necessary and sufficient to specify sequential temporal identity. Switching between the temporal states involves a regulatory network that includes direct interactions between the temporal transcription factors themselves (Kohwi and Doe, 2013).

Advocating developmental biology This article is part of Development's advocacy collection – a series of review articles that make compelling arguments for the field's importance. The series is split into two: one set of articles, including this one, addresses the question ‘What are the big open questions in the field?’ We would argue that there has never been a more exciting time to get involved in developmental biology: incredible new tools mean making fundamental problems are increasingly within reach. A complementary set of articles will ask ‘What has developmental biology ever done for us?’ Together, the articles will provide a collection of case studies looking backwards to the field's achievements and forwards to its potential, and a resource for students, educators, advocates and researchers alike. To see the full collection as it grows, go to http://dev.biologists.org/content/advocating-developmental-biology.

The development of methods that allow the generation of neural progenitors from pluripotent stem cells has shed light on the mechanisms that control the pace of progression through a developmental sequence. The order in which the different types of neurons are generated *in vitro* are the same as *in vivo* (Fig. 1A), and the timing of transitions between cell types is also remarkably similar (Eiraku et al., 2008; Gaspard et al., 2008). Although this does not rule out a role for external signals in modifying the tempo and order of neurogenesis, it emphasises that cell-autonomous mechanisms play a central role in timing developmental progression. Consistent with this, the loss of Ring1B, a component of the polycomb group (PcG) complex, in cortical progenitors results in the prolonged generation of early neuronal subtypes, implicating epigenetic mechanisms in tempo control (Morimoto-Suzki et al., 2014). How this encodes time and what additional mechanisms are involved are poorly understood. Cell division does not seem to be involved, as cell-cycle arrest does not stop the sequence of gene expression in cortical progenitors (Okamoto et al., 2016). Similarly, in the *Drosophila* nerve cord, the sequential gene expression programme continues in G2-arrested neuroblasts (Grosskortenhaus et al., 2005). The development of high-resolution quantitative assays and the ability to perturb the regulatory networks that determine the progression of cell types is likely to yield a better understanding of the underlying mechanisms.

A well established, although somewhat unusual, example of sequential gene expression is the collinear expression of Hox genes: the genes in a Hox cluster are activated sequentially, around the time of gastrulation, and the temporal order of activation matches their relative genomic position within a cluster. The mechanism controlling this temporal collinearity is a long-standing question and new technologies to systematically examine chromatin organisation have recently provided fresh insight into how topologically associated domains (TADs) and cis-regulatory elements contribute to proper expression timing of the Hox genes (Deschamps and Duboule, 2017). Nevertheless, what sets the speed of cluster activation remains unclear.

In general, molecular timers can either count up, by steadily increasing the levels of a critical regulator until it exceeds a threshold, or count down by gradually decreasing the levels of an inhibitor. One example of a count-down timer is the dilution of replication initiation factors by the rapid divisions during early *Xenopus* development. This is proposed to time the mid-blastula transition that initiates zygotic transcription and the slowing of the cell cycle (Collart et al., 2013). Dilution of a finite supply of a factor controlling a cell behaviour thus provides an effective way for an embryo to time a process.

Conversely, a count-up timer appears to determine the seven or eight divisions that oligodendrocyte precursors in the developing rat brain undergo before they differentiate (Raff, 2011). This seems to be controlled, at least in part, by the gradual accumulation of cell cycle inhibitors over time (Dugas et al., 2007). Similarly, in response to nutrient depletion, *Bacillus subtilis* cells undergo several rounds of division before differentiating into spores (Levine et al., 2012). In this case, pulses of expression of the transcription factor Spo0A results in its incremental accumulation to a critical threshold. The pulsatile behaviour appears to make the system more robust than it would be with simple continuous accumulation (Levine and Elowitz, 2014), and hence might represent a strategy exploited by other timer mechanisms. In this context, it is noteworthy that the control of neuronal and glial differentiation in the vertebrate CNS also involve pulses of gene expression (Imayoshi et al., 2013). Whether the reasons for oscillation are similar in these cases awaits further investigation. Nevertheless, understanding the robustness of timers is an important issue, as noise and small variations in initial conditions can have a major effect on accuracy, particularly for timers that operate for long durations.

A well-documented example of a reliable timing mechanism is the generation of somites. These segmental structures form along the anterior-posterior axis of an embryo and later differentiate into muscles, skin and the bones of the vertebral column (Hubaud and Pourquié, 2014). Each new pair of bilaterally symmetrical somites is periodically formed from pre-somitic mesoderm, every ∼2 h in mouse. Linked to the rhythmic generation of somites are oscillations in gene expression in the pre-somitic mesoderm (Fig. 1B). This molecular oscillator – known as the segmentation clock – is driven primarily by the Notch signalling pathway and its transcriptional targets, members of the Hes (Hairy/E(spl)-related) family of transcription factors. The oscillation period closely matches that of somite formation.

What determines the oscillation period of the segmentation clock? Cyclic Hes gene expression is driven by a delayed negative-feedback circuit: Hes activity represses its own expression and Hes protein is unstable so, once repressed, the protein decays rapidly. However, negative feedback without delays results in stable expression. Mathematical models suggest that delays produced by transcription, splicing, translation and the half-lives of gene products influence the oscillation period (Lewis, 2003). Mouse *Hes7*, the most important Hes gene for mouse somitogenesis, has three introns that create a delay in its production. Removing two of these introns speeds up Hes7 production and accelerates Hes7 oscillations; as a result, the period of somitogenesis changes to ∼115 min instead of 127 min (Harima et al., 2012). Indeed, gene length has been suggested as a general biological timer for gene expression (e.g. Kirkconnell et al., 2017), as has the overall size of the genome (Pagel and Johnstone, 1992; Sessions and Larson, 1987); the DNA content of an organism might therefore influence its biological timers. In addition, the nuclear export of mouse Hes7 mRNA to the cytoplasm has been reported to show a ∼20 min delay (Hoyle and Ish-Horowicz, 2013). Thus, the oscillation period appears to result from the combined effect of multiple molecular mechanisms. The recent development of techniques to culture cells and tissues that show oscillatory gene expression *in vitro* raises the possibility of defining the contribution of different molecular processes to controlling the frequency (Matsumiya et al., 2018; Tsiairis and Aulehla, 2016; Webb et al., 2016).

Another well-documented example of a molecular oscillator is the circadian clock: the intricate regulatory feedback loop that gives rise to ∼24 h rhythms in gene expression, physiology and behaviour. The circadian clock has been reported to affect the timing of cell proliferation and differentiation in various adult stem cells (Brown, 2014; Vallone et al., 2007; Weger et al., 2017). This leads to the question of whether the circadian clock also plays a role in embryonic development. Although the circadian clock appears to begin functioning relatively early in development (Dekens and Whitmore, 2008; Yagita et al., 2010), its role, if any, is unclear. Nevertheless, most studies have focused postnatally and it remains a possibility that the circadian clock influences some developmental processes.

## Interspecies differences in developmental time

Although the gene regulatory networks responsible for controlling cell fate and function progress at characteristic rates, these rates differ between species. For example, the generation of different neuronal subtypes in the vertebrate nervous system involves well-defined genetic programmes comprising sequential changes in transcriptional state as cells differentiate from neural progenitors to post-mitotic neurons, and while these programmes are highly conserved across all vertebrates, the timing differs between species (La Manno et al., 2016; van den Ameele et al., 2014). For example, motoneuron generation takes a few days in mouse, but 2-3 weeks in human (Fig. 1C) (Davis-Dusenbery et al., 2014). What explains the difference in duration remains unclear.

Interspecies differences in the durations of distinct neuronal subtype generation are preserved *in vitro* in neural progenitors derived from pluripotent stem cells, suggesting cell-autonomous differences (Barry et al., 2017; van den Ameele et al., 2014). Indeed species-specific patterns are conserved even when progenitors from different primates are cultured together (Otani et al., 2016) or human progenitors transplanted into mouse brains (Espuny-Camacho et al., 2013). A consequence of the differences in rates is that progenitor cell expansion dominates over neurogenesis for a longer period of time in humans (Otani et al., 2016), leading to the proportionally larger cerebral cortices of humans. Moreover, timing mechanisms can affect the balance of proliferation and differentiation to influence the size and cellular composition of a developing tissue. How the frequency of switching between fates is modified between species is not known, although differences in the coding and non-coding regions of genes expressed in cortical progenitors have been documented. These include the presence of hominid-specific genes, which appear to contribute to the enlarged cortex (e.g. Fiddes et al., 2018; Suzuki et al., 2018), as well as differences that affect the spatiotemporal expression and function of conserved genes involved in various aspects of cortical development. These are likely to influence the timing of developmental transitions, but this work is still in its early stages. Recent advances in genomic engineering now offer the opportunity to modify the genome to directly test the importance of species-specific sequences in interspecies differences.

The frequency of somite formation is also species specific: ∼30 min in zebrafish, 90 min in chicken, 2 h in mouse and 5 h in human (Hubaud and Pourquié, 2014). The kinetics of mRNA splicing and export of Hes family members differ between zebrafish, chickens and mice, suggesting that these delays might contribute to the species-specific periods of the segmentation clock (Hoyle and Ish-Horowicz, 2013). Whether these delays are also different among other mammals (e.g. humans) or are sufficient to explain the interspecies period difference remain to be tested (Box 2).

Box 2. An organoid zoo to compare time in different species: a personal case study by Miki Ebisuya When I was a high school student, I read a Japanese book entitled ‘The Time of an Elephant and the Time of a Mouse’ (Motokawa, 1992), which described how larger animals tend to have slower physiological times, including developmental time. I was fascinated by this apparently general rule and wanted to know the molecular mechanisms by which different species displayed different times. However, I thought that studying such interspecies differences would be difficult or impossible, as comparing time in such differently sized animals in a laboratory seemed unrealistic.

Twenty years later, embryonic stem cells (ESCs) and induced pluripotent stem cells (iPSCs) are now available. They can differentiate into different cell types and, with the right culturing conditions, form complex three-dimensional structures such as organoids (Clevers, 2016; Shi et al., 2017). I believe that these *in vitro* approaches offer powerful tools for tackling the ‘elephant time and mouse time’ problem. ESCs and iPSCs have been established from diverse animals, including mouse, rabbit, dog, monkey, human, pig, horse, cow and rhinoceros (Ben-Nun et al., 2015; Ogorevc et al., 2016), and even an elephant iPSC line might one day be possible. Importantly, *in vitro*-differentiated cells and organoids of different species can be compared under the same conditions and experimental setting. Moreover, *in vitro*-differentiated cells are independent of the rest of the body and therefore represent simpler, more manageable systems. In other words, researchers can focus on cell- or tissue-autonomous mechanisms. Finally, several new technologies for quantitative measurements are particularly appropriate *in vitro*, such as single-molecule imaging, high temporal/spatial resolution microscopy and various chemical probes, which makes quantitative comparisons possible.

My group uses the segmentation clock as a model system to investigate interspecies differences in developmental timing. By inducing pre-somitic mesoderm from mouse ESCs, human iPSCs and other mammalian stem cells, we can compare the periods of the segmentation clocks in different animals. Our goal is to uncover the molecular processes in the segmentation clock that are different among these species. Ultimately, we'd like to know how universal the differences are in ‘elephant time and mouse time’ problems.

## Coordinating multiple developmental processes

The evolutionary history of the mammalian cortex demonstrates how changing the tempo and order of development can have profound consequences on the morphology, size or composition of a tissue. However, delaying or accelerating events in one region of a tissue or embryo can also affect other regions by changing spatial relationships or signalling interactions, which alters the relative order of events. Coordinating the timing between different developmental events is therefore necessary for tissue organisation, patterning and morphogenesis. Some of the most poorly understood embryological phenotypes are those arising from mutations in genes that produce embryos with reduced size and developmental delay. This pinpoints the importance of understanding how time is measured.

Different cell types in a developing embryo have different cell cycle lengths and cells divide at different times, resulting in the characteristic regional differences in growth rates. One well-characterised example of this is the *Drosophila* embryo, in which, following the first 13 rapid and synchronous divisions, cells begin to cycle in 25 distinct spatiotemporal domains, each of which has a different but consistent time of division (Foe, 1989). Cell division in the embryo is controlled by the cell cycle phosphatase Cdc25, which activates the cyclin-dependent kinase Cdk1 to initiate mitosis. A combination of spatially restricted transcriptional activators and repressors control expression of Cdc25, and this determines the timing of cell divisions and links cell cycle timing directly to tissue patterning. Moreover, the combinatorial input of these factors is responsible for the precision of the temporal pattern (Momen-Roknabadi et al., 2016). Hence, the regulation of a component of the cell cycle control system provides a means with which to modulate the length of cell cycle in different regions of the tissue. Whether this represents a general mechanism to explain how cell cycle duration is modulated in other tissues remains to be tested.

A notable feature of the early *Drosophila* embryo is the synchrony of the cell divisions within a specific domain. By contrast, in most developing tissues, cells divide asynchronously and the length of the cell cycle in individual cells varies. In addition, growth rate decreases in many tissues as development proceeds (Ricklefs, 2010; West et al., 2001). The molecular and functional basis for these features is unclear, as are the mechanisms that ensure coordination within and between tissues. Quantitative imaging of tissues combined with the new generation of cell cycle reporters (Sakaue-Sawano et al., 2008) are likely to be crucial to address these points.

One way in which the growth of individual tissues can be coordinated in an embryo is by using checkpoints to inhibit premature progression. A well-studied example of this is the metamorphosis of the *Drosophila* larvae. The steroid hormone ecdysone is responsible for triggering pupariation and the onset of metamorphosis that generates the adult fly. This can be delayed for several days if the imaginal discs, which comprise the developing organs of the adult, are damaged or their growth is abnormal. An insulin-like peptide, DILP8, is secreted from imaginal discs with growth abnormalities and activates a systemic neuroendocrine circuit that suppresses ecdysone production (Fig. 1D) (Colombani et al., 2012; Garelli et al., 2012). This ensures that metamorphosis produces adults of the correct proportions and that paired organs, such as wings and legs, are of equal size. However, the mechanisms that control DILP8 expression and how the status of imaginal disc development is sensed and communicated are still under active study (e.g. Boone et al., 2016).

A global mechanism also coordinates *C. elegans* development. After embryogenesis, *C. elegans* progresses through four larvae stages before adulthood. Mutants that display heterochrony – skipping or reiterating specific larval stages – have been identified (Moss, 2007). In total, there are more than 20 genes with heterochronic phenotypes and in many cases mutations in these genes affect all larval tissues, which is indicative of a global mechanism. The genes responsible encode microRNAs and the factors that control their expression. Together, these constitute a regulatory network known as the heterochronic pathway. The expression of specific members of the heterochronic pathway define each larval stage and the regulatory interactions between these genes act as switches that determine transitions between stages. Hence, in heterochronic mutants, developmental events are shifted and the sequence disrupted. Although these ensure the correct sequence of development and coordinate developmental progression across the larva, how the precise timing of switching is achieved remains unclear. Moreover, whether global or systemic control mechanisms synchronise developmental events in animals that do not undergo metamorphosis, including many vertebrates, remains unclear (see, for example, Rosello-Diez et al., 2017 preprint).

It seems likely that the developmental tempo for different processes and in different tissues must change proportionally with the growth of the embryo. For example, the period of the segmentation clock needs to be consistent with the rate of embryo elongation in order for the correct number and size of somites to form in each species. The number of somites formed in a snake embryo is much larger than that in other amniotes: ∼300 somites in snake compared with 65 in mouse. This is because the segmentation clock is much faster relative to the embryo elongation rate in snake compared with other species (Gomez et al., 2008). Similarly, accelerating the mouse segmentation clock by genetic manipulation results in a increased number of (and a smaller size of) somites (Harima et al., 2012). A consequence of this need for temporal coordination is that changing the rate of one developmental mechanism has knock-on consequences for other, apparently unconnected, processes. This type of constraint might explain why embryos of different species tend to have characteristic developmental times. It is notable, for example, that disparate mechanisms such as the segmentation clock and motoneuron differentiation take two to three times longer in human than in mouse.

What could explain global scaling properties? Temperature is well known to affect biological processes. This might be relevant in ectotherms (fish, reptiles, insects, etc.). For example, the period of the segmentation clock of zebrafish embryos changes more than threefold across a 10°C temperature range (20-30°C), whereas the overall length of embryos is constant (Schroter et al., 2008). This exemplifies how temperature-induced alterations in growth rate are matched by changes in the oscillation period. However, for endotherms, and mammals that develop *in utero*, temperature is relatively constant and unlikely to be a major contributing factor to setting the pace of development. Moreover, the segmentation clock of zebrafish embryos, at the typical temperature of 28°C, is more than four times faster than the mouse segmentation clock which operates in tissue that is kept at ∼37°C, indicating that temperature is not solely responsible for the differences in tempo.

Similar to temperature, metabolic rate – the rate of energy use by cells – also has global effects on cellular and molecular processes, and has been suggested to provide a general explanation for allometric growth and timing (West et al., 2001): larger animals have correspondingly slower metabolic rates. Whether and how metabolic rate affects specific molecular and developmental processes, such as the dynamics of a gene regulatory network, is not known. In this context, it is notable that *clk-1* mutant *C. elegans* have lengthened developmental stages and cell cycle, as well as changes in the timing of behaviours such as pharyngeal pumping, defecation and locomotion (Branicky et al., 2000; Wong et al., 1995). *clk-1* encodes a mitochondrial enzyme that is necessary for the biosynthesis of ubiquinone, an obligate electron transporter in the mitochondrial electron transport chain, and *clk-1* mutants have reduced, albeit modestly so, mitochondrial function (Felkai et al., 1999). Intriguingly, mice lacking *Clk1* are developmentally delayed by midgestation (Levavasseur et al., 2001; Nakai et al., 2001). Detailed analysis will be needed to determine whether these defects affect timing mechanisms and how changes in metabolic rate might be linked to the rate of development. Identifying further genetic mutants or experimental perturbations that affect global developmental rates as well as visualizing metabolic rates in living tissues (Imamura et al., 2009; Tantama et al., 2013) might provide further insight.

## Conclusions

Taken together, studies from a range of systems are beginning to shed light on the molecular and genetic mechanisms that encode and interpret developmental time. Comparisons between species and results from different tissues are identifying the players and mechanisms that account for the order and tempo of developmental events. New reagents, particularly novel imaging reporters, and the development of new techniques, such as *in vitro* tissue culture methods, are offering a fresh perspective on this long-standing problem. This is sharpening the focus on the underlying principles and relevant questions, answers to which will provide insight into a fundamental aspect of embryo development.
